# Association between left-displaced abomasum corrected with 2-step laparoscopic abomasopexy and milk production in a commercial dairy farm in Italy

**DOI:** 10.1186/s13620-018-0132-2

**Published:** 2018-10-09

**Authors:** Filippo Fiore, Daniele Musina, Raffaella Cocco, Alessandro Di Cerbo, Nicoletta Spissu

**Affiliations:** 1 Department of Veterinary Medicine, University of Sassari, Via Vienna 2, 07100 Sassari, IT Italy; 2 Freelance veterinarian, Loc. Perdas Arbas, 08100 Nuoro, Italy; 3 Department of Life Sciences, University of Modena and Reggio Emilia, Modena, Italy; 4 Department of Medical, Oral and Biotechnological Sciences, Dental School, University G. d’Annunzio of Chieti-Pescara, Chieti, Italy

**Keywords:** Dairy cow, Left displacement of abomasum, Milk yield

## Abstract

**Background:**

Left displacement of the abomasum (LDA) is a condition of dairy cows that causes huge economic losses. The aim of the study was to evaluate the effect of LDA after on-farm correction by the 2-step laparoscopic abomasopexy on milk production based on 305-d milk yield on a commercial dairy farm in Italy.The study was performed between January 2011 and January 2014 on 58 Holstein Friesian cattle with left displacement of the abomasum in a commercial dairy farm in the farmland of Ozieri, Sardinia (Italy). Each cow underwent a 2-step laparoscopic abomasopexy performed by the same veterinarian. Each case was matched with a control herdmate by age, parity and calving date. Cows with LDA and healthy control cows also had a similar 305-d milk yield in the previous lactation. Data on milk production were collected using a dairy herd management software programme (Afimilk®, Afimilk Ltd., Israel). The 305-d lactation yield was obtained from the sum of daily milk yields for each cow. An unpaired Student’s t-test was used to compare changes in milk production, mean fat and protein percentage of cases and controls before and after surgical procedure.

**Results:**

Data from 4 cows were excluded from the analysis due to post-surgical complications. 54 cases and 54 control cows participated in the study. We found that milk production significantly decreased from a baseline of 12,295 ± 1690 kg to 11,165 ± 1989 kg in the affected lactation. Conversely, a significant increase was observed for mean fat and protein percentage during lactation in case cows.

**Conclusions:**

In the present study cows with left displacement of the abomasum corrected with 2-step laparoscopic abomasopexy produced less milk than their control herdmates. Each case and control pair in the present study came from the same farm in order to eliminate farm to farm differences in management, housing, season, etc. However, this limits the validity of our data to the specific situation described here.

## Background

Left displacement of the abomasum (LDA) is a condition that occurs primarily in high producing postpartum dairy cows [1]. Normally the abomasum is situated in the ventral part of the abdomen and is filled with fluid. LDA arises when the abomasum becomes enlarged primarily due to gas distention and is mechanically displaced from its normal position to the left side of the abdominal cavity, between the rumen and the left lateral abdominal wall. The rumen usually descends to trap the abomasum in this abnormal position [2]. Risk factors for development of LDA include breed, age and season [3], as well as nutritional and management factors [4]. Dairy cows with periparturient disorders such as ketosis, dystocia, stillbirth, metritis and hypocalcaemia are more likely to develop the condition [5]. Many different treatment options have been developed for the correction of LDA, from dietary interventions to open and closed surgical treatments to fix the abomasum [6]. Commonly used open surgical techniques include omentopexy via laparotomy in the right paralumbar fossa, abomasopexy via ventral paramedian approach, and left paralumbar fossa abomasopexy. Closed surgical techniques include percutaneous fixation by blind-tack suture or toggle-pin [7]. Percutaneous fixation is the most rapid technique but has the disadvantage that it is performed in a blind fashion by the veterinary surgeon, so there is a risk of stitching the wrong organ or of trapping abdominal contents within the “pexy” site [8]. The 2-step laparoscopy-guided fixation of the abomasum is a more recent technique [9] that combines the advantages of a traditional open surgical technique (good visual inspection, therapeutic safety) with the rapidity and shortened recovery times of percutaneous fixation methods [10]. An important issue to consider when deciding to correct LDA is the outcome following surgery, in particular the extent of milk losses attributable to the disease and its correction. In order to have an advantageous correction, the cow has to return to her previous level of productivity, avoiding further economic losses [8]. In the past decades several authors investigated the effects of LDA on milk production using different approaches to treat it and quantify milk losses [11]. More recently, other studies evaluated milk losses due to LDA in cows treated with the 2-step laparoscopy-guided abomasopexy [6, 10, 12]. In detail, Seeger et al. considered only the short term outcome of the treatment, while in the other two studies the subjects came from different farms, had concurrent diseases and the correction was performed by different veterinarians. To the best of our knowledge, no study has been conducted on 305 day milk yield following LDA correction using the 2-step laparoscopic abomasopexy performed by the same veterinarian, in farm settings, on cows coming from the same farm and free from other observable concurrent diseases than LDA.

The objective of our study was therefore to evaluate the effect of LDA after on-farm correction by the 2-step laparoscopic abomasopexy on milk production in a commercial dairy farm in Italy.

## Method

### Animals and management

This study was performed between January 2011 and January 2014 on cows with LDA, diagnosed by a veterinarian from the University of Sassari, in a commercial dairy farm in the north of Sardinia (Italy). The farm consisted of 280 lactating Holstein cows, housed in free-stall barns and milked twice a day. Grouping of cows into different pens was based on stage of lactation. Dry cows were fed two total mixed rations (TMR), one during the first 30–40 days of the dry period, and a “close up” TMR during the last 21 days of gestation. All lactating cows were fed twice daily the same TMR formulated by the herd nutritionist. The TMR met or exceeded National Research Council requirements for 650-kg lactating cows producing 35 kg/day of milk with 3.5% fat and 3.1% protein [13].

In total, 373 Holstein dairy cows (125 primiparous and 248 multiparous cows) calved in the study period and were monitored twice a week for one month and on call if the farmer noticed any health problem.

Diagnosis of LDA was based upon the presence of an acute ping sound on auscultation and percussion of the left side of the abdomen. Inclusion criteria were: multiparous cows with an LDA independent of the degree of displacement and duration of clinical signs, less than 30 days in milk (DIM) at the time of the diagnosis, absence of concurrent diseases (i.e. clinical metritis, retained fetal membranes, hypocalcemia, ketosis, mastitis, lameness).

Clinical metritis was defined as cows having an abnormally enlarged uterus, a fetid, watery, reddish brown uterine discharge with fever (> 39.5 °C) and presence of signs of systemic illness (decreased milk production, dullness, or other signs of toxemia) within 21 days postpartum.

Retention of placenta was defined as the failure to expel fetal membranes within 24 h after parturition.

If the blood Ca concentration was ≤ 2 mmol/l (8 mg/dl), it was defined as hypocalcemia.

Cows with BHB ≥ 1.2 mmol/l, but no clinical signs (off feed, decreased milk yield) were considered to be in a state of subclinical ketosis.

Clinical mastitis cases were characterized by the presence of abnormal milk or by signs of inflammation in one or more quarters.

Lameness can be defined as the clinical manifestation of painful disorders, mainly related to the locomotor system, resulting in impaired movement or deviation from normal gait or posture.

Exclusion criteria included surgical and postsurgical complications, any other disease during a complete lactation (305 days), including cows with infertility resulting in them being open for > 100 days. After confirmation of eligibility, 58 multiparous cows with LDA were included. Each case was matched with a control herd mate, based on age (± 1 month), parity and calving date (± 1 month). Cases and controls for multiparous cows also had a similar 305-d milk yield (± 200 kg) in the previous lactation. The animals were followed during a complete lactation (305 days). Herd health was evaluated twice a week by a veterinarian from the University of Sassari. Data concerning insemination date(s) and 305 day milk production in the current lactation were collected for all the animals.

### Laparoscopic technique for LDA correction

Each cow underwent a 2-step laparoscopic abomasopexy within 24 h after the diagnosis by the same veterinarian (Janowitz, 1998). The procedure was performed in a clean pen rich in straw bedding. The cases were not sedated during the procedure. During the second step of the correction, each animal was cast with ropes and moved first in right lateral recumbency and then turned clockwise into full dorsal recumbency. Hay bales were used to support the shoulders and front and hind limbs were tied to an extended position. No postoperative treatment was administered, and immediately after correction the cows returned in the lactating group. The gauze bandage and knot were removed approximately 28 days after surgery.

### Milk data collection

Data on milk production were collected from a computerized dairy herd management software programme (Afimilk®). The 305-day lactation yield was obtained from the sum of daily milk yields for each cow.

### Statistical analysis

Data were analyzed using GraphPad Prism 6 software (GraphPad Software, Inc., La Jolla, CA, USA). All data are presented as the means ± standard error of the mean and were first checked for normality using the D’Agostino-Pearson normality test. An unpaired Student’s t-test was used to compare changes in milk production, mean fat and protein percentage of treated and control cows during the entire study period before and after treatment. A ∗*p* < 0.05 was considered significant.

## Results

During the study period, 88 cows were diagnosed with LDA. Among them, 16/88 (18.2%) were excluded as they were primiparous. Of the remaining 72 multiparous cows, 14/72 (19.4%) were excluded due to concurrent diseases. A total of 58 multiparous cows with LDA underwent surgical correction. Data from 2 cows were excluded from the analysis due to post-surgical complications including severe peritonitis and relapse due to breakage of toggle-pin suture. Two other cows developed mild to severe bronchopneumonia and were culled before the end of the study. Complete data were collected for 54 cases and 54 control cows from the same herd.

The median interval between calving and the diagnosis was 11.4 ± 6.8 with a range from 1 to 29 days. Of the 54 cases, 13 were in parity 2 (24.1%), 17 were in parity 3 (31.5%) and 24 were in parity 4 (44.4%) (Table 1).

**Table 1:** Distribution of 54 cows with LDA as defined by lactation number

Lactation number	No. of cases	Percentage of cases (%)
2	13	24.1
3	17	31.5
4	24	44.4

The interval from parturition to LDA surgery was ≤ 7 days in 16 cows (29.6%), between 8 and 14 days in 19 cows (35.2%), between 15 and 21 days in 15 cows (27.8%) and between 22 and 30 in 4 cows (7.4%) (Table 2).

**Table 2:** Time interval from parturition to LDA surgery in 54 cows

Time interval (days)	No. of cases	Percentage of cases (%)
≤ 7	16	29.6
8–14	19	35.2
15–21	15	27.8
22–30	4	7.4

**Fig. 1:**
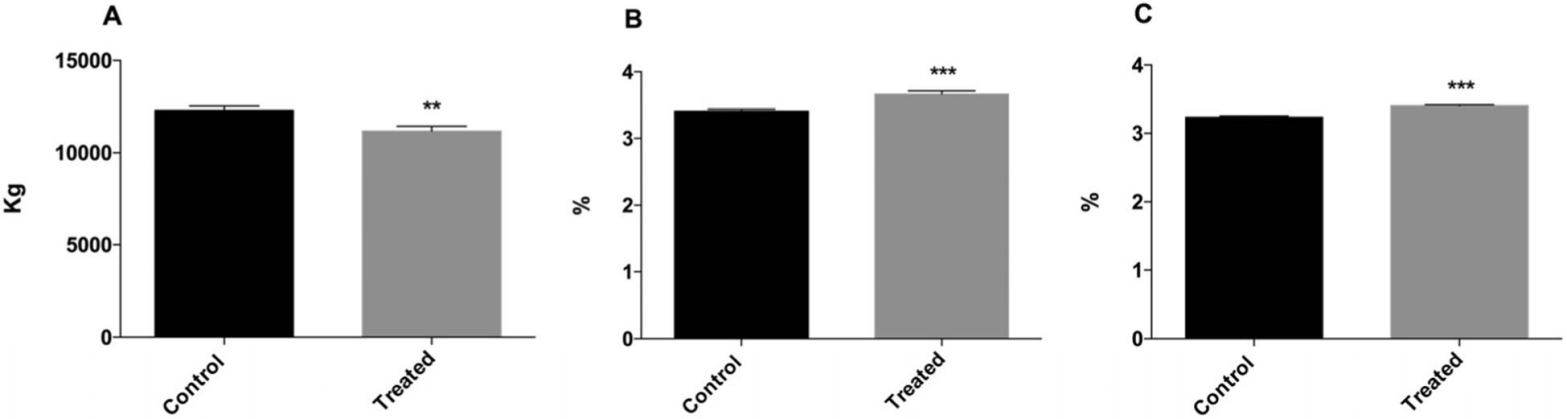
Graphical representation of (**a**) milk production during lactation, (**b**) mean fat production during lactation, and (**c**) mean protein production in multiparous cows belonging to control and treated groups

Milk production in the study group was from 11,165 ± 1989 kg (**p < 0.01) whereas the control group produced 12,295 ± 1690 kg. Conversely, a significant increase in mean fat and protein percentage from a baseline of 3.40 ± 0.23% to 3.66 ± 0.36% and from of 3.23 ± 0.14% to 3.40 ± 0.11% was observed, respectively (***p < 0.001) (Fig. 1).

## Discussion

In the present study, LDA occurred most frequently in the first and second week postpartum (29.6% and 35.2%, respectively) and in parity 4 cows (44.4%), which is in accordance with other studies [6, 14].

In our study, LDA was associated with decreased milk production during the affected lactation, which is in accordance with the results of several other studies [2, 15–21]. In detail, the results of the present study show an overall loss in milk yield in the study group compared with its control group (11,165 vs. 12,296 kg) during a whole lactation (305 days). Reported milk production losses caused by LDA vary among studies, ranging from 557 to up to 1000 kg [2, 20].

The severity of the losses is due to the fact that LDA often develops in early lactation, affecting peak yield [16] as well as the digestive system and metabolism [19]. At the time of the diagnosis the subjects were on average 11.4 ± 6.8 DIM.

Part of the milk losses is likely to occur before the diagnosis of the disease is made [1, 16, 18, 22]. Several authors provided evidence that milk losses due to LDA occur as soon as 10 days before diagnosis [23] and they account for 30% of 305-d losses [16]. Typically, all cows in the immediate post parturient period experience an energy and nutrient deficit, whereby cows experiencing LDA lower their feed intake long before the occurrence of disease [24]. The pre-clinical effects of the disorder include decreased DMI for several days prior to clinical diagnosis, thus lowering daily milk yield [4].

Although it is well known that surgical correction may have additional depressing effects on milk yield [24], in this study we used the 2-step laparoscopic abomasopexy technique, which is minimally invasive and seems to result in improved convalescence during the immediate post-operative period, resulting in an earlier increase in milk yield when compared to other surgical techniques [10, 19, 25]. For these reasons, in our opinion the technique contributed only marginally to the depression in milk yield. An analgesic drug was not administered to the patients after surgery, because in the authors’ knowledge, the benefits of NSAIDs in minimally invasive procedures to correct abomasal displacement have not been objectively demonstrated [26] and because the laparoscopic procedure itself is associated with less postoperative pain [10]. However, pain associated with the procedure would also have accounted for some of the milk losses of the cases.

Furthermore, none of the cows with LDA in this study had concurrent diseases, in accordance to our exclusion criteria. The decline in milk yield registered differs from data reported in other studies in which LDA was associated to other diseases [6, 12] and it is likely attributable to the fact that these cows could have been affected for several days before the diagnosis and that the disease occurred in early post-partum, affecting subsequent peak yield. In the farm subject of the present study, LDA was likely related to inadequate nutritional management of cows during the transition period and after LDA correction, so this may have limited the recovery in mik yield of the cases.

In addition to the milk losses, we could show a significant increase in milk fat and milk protein percentage in the LDA group. Higher milk fat content for LDA cows might have been caused by the lower milk production in association with greater body weight losses due to the disease, which are known to contribute to increase milk fat content [27]. In fact, a study reported the existence of a mechanism that increases milk fat content when yield is compromised [28]. As regards the increase in milk protein percentage, it may be directly related to the dilution, by the negative correlation between milk yield and solid concentration [29].

## Conclusions

In the present study cows with LDA corrected with 2-step laparoscopic abomasopexy produced less milk than their control herd mates.

In our opinion these results reflect the impact of the disease itself and of its correction on milk yield, as LDA was not complicated by the effect of concurrent diseases and because case-control comparison was made on the basis of parity, calving date and production in the lactation preceding the LDA affected lactation, to minimise bias. Each case and control pair in the present study came from the same farm in order to eliminate farm to farm differences in management, housing, season, etc. This does, however, limit the validity of our data to the specific situation described here.
